# Facilitating Access and Adherence to Physical Activity and Exercise for Service Users With Neurological Conditions in the Community: A Service Evaluation

**DOI:** 10.1111/jep.70146

**Published:** 2025-07-24

**Authors:** Caroline Appel, James Alexander, Simon Conroy, Cherry Kilbride

**Affiliations:** ^1^ Camden Neurology and Stroke Service Central North West London NHS Foundation Trust London UK; ^2^ Lifelong Health and Ageing University College London, Central North West London NHS Foundation Trust London UK; ^3^ Department of Health Sciences Brunel University of London Uxbridge UK

**Keywords:** co‐production, exercise, health, health services research, neurological conditions, physical activity, service evaluation

## Abstract

**Rationale:**

Many service users with neurological conditions do not meet the recommended physical activity requirements. Cultivating early and ongoing access to physical activity and exercise opportunities is vital to improve or maintain function and general health in this vulnerable group.

**Aim:**

To evaluate the impact of a pathway that aimed to facilitate access and adherence to physical activity and exercise for service users with neurological conditions.

**Methods:**

A London‐based NHS healthcare team providing community neurorehabilitation developed a pathway in co‐production with public health, local authority, third sector parties and service users to facilitate physical activity and exercise opportunities. First, NHS neurophysiotherapists offered a bespoke programme on exercise, physical activity and education to service users for up to 12 weeks. The pathway continued in local gyms, supported by a fitness instructor, for at least a further 12 weeks. Using a pre–post design, outcomes relating to function, strength and physical activity were recorded at baseline, 6–12 weeks (health care) and 6 weeks later (telephone survey after transition to local gyms). Data analysis was descriptive.

**Results:**

Thirty‐five service users (20 men), mean (SD) age 60 (15), with a range of neurological conditions, were eligible and included. Ten participants dropped out: eight (23%) for medical reasons, two (6%) for other reasons. Due to the COVID‐19 pandemic, four (11%) service users could not transition when facilities closed in March 2020. Analysis showed potential beneficial effects on function, strength and physical activity for service users as well as reduced waiting times to access the NHS and local gyms.

**Conclusion:**

Outcomes suggested the pathway enabled service users to access and adhere to physical activity and exercise following neurorehabilitation. This evaluation included small numbers but could inform service development and future studies.

**Abbreviations**
10MWT: Ten‐metre walk test
BP: blood pressure
GP: general practitioners
IQR: interquartile range
MOEES: Multidimensional Outcome Expectations for Exercise Scale
MS: multiple sclerosis
NHS: National Health Service
PAVS: Physical Activity Vital Sign
PD: Parkinson's disease
SD: standard deviation
STS: sit‐to‐stand

## 1 Introduction

People living with chronic diseases are at greater health risk from non‐communicable diseases because of their physical inactivity [1]. People living with neurological conditions are particularly vulnerable to being physically inactive and leading sedentary lifestyles [1, 2, 3], both of which are associated with high health care costs [4]. Sedentary behaviour is defined as: ‘any waking behaviour characterised by an energy expenditure ≤ 1.5 metabolic equivalents while in a sitting or reclining posture’ [5]. Recommendations are to replace sedentary behaviour with physical activity of any intensity but preferably moderate to vigorous intensity [6, 7, 8, 9, 10, 11] or with exercise, a subset of physical activity that is planned, structured and repetitive, aiming to improve or maintain physical fitness [12]. During moderate intensity physical activity or exercise, breathing should increase, and during vigorous intensity, breathing is fast [8]. WHO guidelines on physical activity and sedentary behaviour for adults living with a disability including stroke, multiple sclerosis (MS), Parkinson's disease (PD) and spinal cord injury recommend at least 150–300 min of moderate or 75–150 min of vigorous intensity aerobic physical activity each week as well as muscle strengthening activities at moderate or greater intensity involving all major muscle groups twice weekly [6]. In the absence of specific physical activity guidance for certain neurological conditions, for example, traumatic brain injuries, it is suggested that indirect evidence from other neurological populations with similar cognitive, behavioural and physical impairments is followed [13].

In addition to the benefits to general health, physical activity and exercise may also have disease‐modifying effects as investigated in some neurological populations. After stroke, for example, exercise delivered at high enough intensity of at least 45 min [10] up to 3 h each day [14, 15] can facilitate brain re‐organisation and improve clinical outcomes. In early PD, maintenance of high (moderate to vigorous) intensity regular physical activity levels and exercise habits was associated with better clinical course of the disease [16], specifically reduced severity of motor signs and improved quality of life [17]. For service users with MS, the recommendation is to empower self‐management and implementation of a physically active lifestyle to reduce disability in addition to early intervention with disease‐modifying treatment [18, 19, 20, 21]. Exercise was recommended as safe and not associated with MS relapses [22, 23, 24].

Despite strong evidence of benefit, adherence to physical activity and exercise can be challenging for many, especially those who find moving difficult. The evidence for some neurological populations suggests that barriers arise from personal factors as well as the environment, which challenge perceptions of safety and confidence to exercise [25, 26]. In service users with PD, adherence to exercise could improve through: (i) removing person‐specific barriers and influencing motivators [27]; while in stroke survivors, (ii) social networks [28], (iii) formal programmes [28] and (iv) community participation [29] have been found to facilitate physical activity and exercise participation.

Exercise programmes developed with input from service users may be better as these programmes are more relevant to users and could, therefore, facilitate access and adherence to programmes. Examples include FAME [30] and I‐REBOUND [31], two programmes that facilitate physical activity and healthy living after stroke. Locally, service users struggled to transition from supported National Health Service (NHS) exercise input to exercising in the community. For example, after discharge from the NHS exercise groups, service users were re‐referred after a short period for further exercise provision. This contributed to the lengthening of waiting lists and service users' deconditioning, affecting their physical abilities and quality of life. The NeuroActive pathway was co‐produced with service users and local providers to support the transition of service users from the NHS to community exercise schemes. First, this paper aims to describe the co‐production process of the pathway, and second, to evaluate the pathway on (i) performance and (ii) feasibility of service user adherence and outcomes on function, strength and physical activity measures.

## 2 Methods

### 2.1 Aim One: Co‐Production Process

The term co‐production in this paper refers to a consultation and collaboration process with service users and providers as equal partners [32, 33]. Engaging those with lived experience in co‐production could be beneficial to individuals through improved health outcomes as well as for society as a whole through improving healthcare policy and practice [34, 35]. In this project, co‐production was embedded through elements of co‐design, co‐decision making, co‐delivery and co‐evaluation [36].

#### 2.1.1 Co‐Design, Co‐Decision Making, Co‐Delivery of the New Pathway

Service users with neurological conditions were unable to consistently access local gyms or groups that offered physical activity and exercise; this limited the transition from NHS to public services, as local services were not catering to their specific needs. To improve this, an engagement event was organised in 2016 to facilitate information sharing between service users and providers about barriers and facilitators to local exercise schemes that existed and what improvements could be made. The event was organised in a central and accessible location by the Business and Transformation Manager, A.P., and neurophysiotherapist, C.A. Invitations were sent via email/phone/face‐to‐face to people with an interest in providing or receiving physical activity and exercise opportunities locally. Service users and providers such as Better Gym (a non‐profit charitable social enterprise organisation, Greenwich Leisure Limited) and Charities were encouraged to invite those within their network. Light refreshments were provided as well as re‐imbursement for service users' travel costs. Taxis and support for service users to attend were offered.

#### 2.1.2 Co‐Evaluation

Four service users who had transitioned to Better Gym 3–6 months earlier were interviewed to evaluate the delivery of the new pathway. A.P. conducted informal face‐to‐face interviews at a convenient location and explored individual experiences of the pathway by using an open questioning technique. NHS physiotherapists and local providers, including fitness instructors, also met quarterly to share experiences about the delivery of the pathway to ensure it remained true to its initial co‐design with service users. Continued informal feedback about the pathway from service users and how to best collaborate with general practitioners (GPs) was also discussed at these meetings.

### 2.2 Aim Two: Evaluation of the Pathway

#### 2.2.1 Service Users

Service users included those:

- referred to the NeuroActive service between January 2019 and January 2020 for physical activity and exercise
- with a neurological condition
- with a goal to increase or maintain strength and fitness
- with a goal to transition to local exercise options
- of 18 years old or above
- who could attend as an outpatient: travel and mobilise for short distances independently or with the support of a carer.

#### 2.2.2 NeuroActive Intervention

##### 2.2.2.1 NHS and Better Gym Settings

Service users entering the pathway started the NeuroActive intervention in an NHS gym equipped with bikes, a treadmill, a ‘multi gym’, as well as neuro‐specific equipment, including parallel bars and MOTOmed machines offering assisted pedalling suited for all abilities. Better Gym purchased MOTOmed machines as well as strengthening and cardio equipment that was inclusive to access. The equipment accommodated gradual weight increases and visual feedback of performance, making it easier to operate for all users. They had parts that could be removed to allow access for wheelchair users and had back supports or additional straps on rowing machines and bikes to support those with reduced balance and/or strength. These adaptations were low‐cost and made the equipment more inclusive to all users. The Better Gym environment had step‐free access and was set up with more space around machines to accommodate wheelchair users to manoeuvre and transfer onto these machines. Accessible toilets and hoists were available in the changing rooms.

##### 2.2.2.2 Intervention

The NeuroActive intervention, delivered by a neurophysiotherapist and a rehabilitation assistant, entailed a personalised exercise programme delivered in a small group as well as advice on physical activity, exercise, pacing and self‐management. The sessions of 1 h started with a warm‐up and ended with a cool‐down. Most individual programmes were aerobic and strength‐based, typically including cycling (upright or recumbent bike) and/or walking on a treadmill or gym floor (using parallel bars if required) followed by a lower limb and/or arm and/or trunk‐strengthening routine using machines, weights, exercise bands or simply active movements. For wheelchair users and those with severe weakness and/or fatigue, the MOTOmed machine was a popular modification to train arms and legs at varying speeds and resistance. If not enough strength can be generated, this cycle could assist service users with arm or leg pedalling. The recommended intensity of aerobic exercise was moderate (increased breathing) or vigorous (fast breathing) [8]. For some service users, this was their first exposure to exercise and care was taken to build confidence before focusing on intensity. Education and pacing were central to the programme, as well as ensuring adherence to a new routine. Once comfortable in the NHS gym, service users were asked to conduct aerobic exercise at a target heart rate of 60%–85% of age predicted maximum heart rate (220‐age) and conduct strengthening exercise to 3× 8–10 repetitions at 70% of one maximum repetition resistance (6, 11; Tables 1 and 2). Exercises were modified following physiotherapy assessment as required to ensure safety within the small group of five service users. This could mean using the more supportive MOTOmed machine over an upright bike or walking ‘over land’ rather than on a treadmill, or choosing seated arm strengthening rather than standing.

**Table 1:** Service user characteristics.

Characteristics of 35 service users	Value
Sex, *n* (%)	
Female	15 (43)
Male	20 (57)
Ethnicity	
White British background	22 (63)
White with other backgrounds	4 (11)
Asian (1 Chinese and 2 other Asian backgrounds)	3 (9)
Black (1 African, 1 Caribbean, 2 other black backgrounds)	4 (11)
Unknown background	2 (6)
Age (years), mean (SD)	60 (15)
Neurological condition, *n* (%)	
Stroke	11 (31)
Multiple sclerosis	6 (17)
Parkinson's disease	5 (14)
Other	5 (14)
Spinal cord injury	4 (11)
Traumatic brain injury	4 (11)
Co‐morbidities (> 3), *n* (%)	33 (94)
Able to walk, *n* (%)	27 (77)

Abbreviations: *n*, number; SD, standard deviation.

**Table 2:** Service user outcomes at the start and end of the NeuroActive NHS intervention.

Outcome measures	Baseline (Mean/SD) IQR	End (Mean/SD) IQR	Change (Mean/SD) IQR	Nr/35 (%)^a^
MOEES^b^	61.9 (8.9) 55.7–69.5	62.8 (8.3) 60.0–69.2	0.8 (8.3) −4.5–6.7	20/35 (57)
BP^c^ systolic	130.2 (17.5) 117.0–138.0	129.2 (14.7) 118.0–137.0	−1.0 (16.2) −13.0–10.0	19/35 (54)
BP^c^ diastolic	82.3 (9.0) 78.0–89.0	77.2 (9.6) 70.0–82.0	−5.1 (7.9) −11.0–1.0	19/35 (54)
10MWT^d^–Normal	12.4 (5.5) 7.7–17.8	11.6 (4.9) 7.9–15.7	−0.8 (2.6) −2.8–0.7	17/35 (49)
10MWT^d^–Fast	9.1 (4.6) 5.6–13.8	8.3 (3.4) 5.7–11.1	−0.5 (1.5) −1.6–0.1	16/35 (46)
STS^e^	9.4 (5.9) 5.0–13.0	10.1 (4.6) 7.0–13.0	0.7 (3.7) −1.0–3.0	23/35 (66)
Hand Grip Dynamometry right (kg^f^)	30.1 (13.6) 18.0–41.3	32.9 (13.6) 20.0–42.5	2.7 (3.8) −0.1–6.2)	17/35 (49)
Hand Grip Dynamometry left (kg^f^)	27.1 (11.0) 18.0–36.0	31.5 (11.3) 24.2–39.7	4.7 (5.0) 0.7–7.0	20/35 (57)
PAVS^g^	38.6 (119.1) 0.0–30.0	177.9 (420.6) 0.0–52.5	139.3 (383.2) 0.0–52.5	14/21 (67)

^a^ Number of service users (percentage) with entry and discharge data for each outcome measure.

^b^ Multidimensional Outcome Expectations for Exercise Scale (max score 75).

^c^ Blood pressure (mm Hg).

^d^ Ten‐metre walk test (s).

^e^ Number of sit‐to‐stands in 30 s.

^f^ Kilograms.

^g^ Physical Activity Vital Sign (min).

Time to transition to a local gym was individualised according to service user need up to a maximum of 12 weeks. Local gym choice and time of transition were discussed at baseline as a goal to work towards. During transition physiotherapists would accompany service users to Better Gym and two joint sessions with fitness instructor and physiotherapist would ensure safe transition and agreement about the future programme.

#### 2.2.3 Data Collection

Data collected to evaluate (i) performance and (ii) feasibility of the pathway (Aim 2) were logged on an Excel spreadsheet at entry and discharge from the NHS Physiotherapy Service (baseline, between six and 12 weeks) and at follow‐up (6 weeks later) in the community (when transitioned). Weekly attendance and date of transition were logged as well.

Performance data reflecting flow through the service and service user adherence (i) were:

a. Response time of referrals to the start of the: (i) Neuroactive programme and (ii) local gym programme (days);
b. Number of service users transitioning from the NeuroActive gym to local gyms;
c. Number of service users not attending or dropping out, including reasons why.

Feasibility of collecting service users' outcome data (ii) was:

- Number of NeuroActive sessions attended to assess adherence;
- Physical Activity Vital Sign (PAVS) to assess the number of minutes of physical activity completed in the last 7 days (1. On average, how many days per week do you engage in moderate to vigorous physical activity (like a brisk walk)? _____ days; 2. On average, how many minutes do you engage in physical activity at this level? _____ minutes. Total minutes per week of physical activity (multiply #1 by #2) _____ minutes per week). This measure was added to the protocol following the first quarterly review by physiotherapists [37, 38];
- Ten‐metre walk test (10MWT) (in seconds) to assess walking speed [39, 40];
- Six‐minute walk or equivalent for wheelchair users: 6‐min push test (total distance [m] of walking or self‐propulsion in 6 min) to assess endurance [39, 40];
- Sit‐to‐stand (STS) in 30 s (number of stands completed) to assess leg strength and endurance [41];
- Blood pressure (BP) (mm Hg);
- Multidimensional Outcome Expectations for Exercise Scale (MOEES) (on a Likert scale of 5 with a total score of 75 to assess expectations; higher scores are indicative of higher levels of outcome expectations for exercise) [42, 43];
- Dynamometry grip strength (kg) to assess strength [44].

Data collection was completed by NeuroActive physiotherapists working in a Central London NHS therapy gym between January 2019 and March 2020. NeuroActive physiotherapists had agreed on this set of outcome measures to use as appropriate per service user and were trained to undertake them. Following baseline data collection, data collected at discharge from the NHS were at flexible time points (between six and 12 weeks) to match individual needs and the length of time needed in the NHS gym before transitioning to local gyms. Physiotherapists called service users who did not attend twice in a row to enquire about their well‐being and discuss if they could continue. If unable, they would drop out of the NHS programme and were encouraged to re‐refer themselves. Those who completed the NHS programme and transitioned to a local gym received a follow‐up phone call 6 weeks later to discuss attendance and record the PAVS. Local gyms did not record performance, attendance or physical data. Quarterly meetings and training facilitated adherence to protocol procedures

Ethical approval was not required as per Health Research Authority and Trust governance procedures, as participants were: (i) not randomised to different groups, (ii) treatment was not changed from accepted standards and (iii) the service evaluation was not designed to produce generalisable results [45].

#### 2.2.4 Data Analysis

Continuous variables are presented as mean and standard deviation (SD) and categorical data as numbers (*n*) and percentages (%). All data were included to describe the cohort in terms of demographics and pathway performance. Outcome data only for those service users with complete pre‐ and post‐measures are shown along with change scores.

## 3 Results

### 3.1 Aim One: Co‐Production Process (2016–2019)

#### 3.1.1 Co‐Design, Co‐Decision Making, Co‐Delivery of the New Pathway

The engagement event lasted for 3 h and was attended by the following: 3 service users, 1 private physiotherapist, 10 NHS neurophysiotherapists (local community and hospital‐based including 3 from external boroughs), 1 occupational therapist, 2 rehabilitation assistants, 1 social worker who had set up a community centre offering supported exercise opportunities in an external Borough, 3 local authority and third sector representatives responsible for providing physical activity and exercise opportunities to local residents with neurological conditions and 3 local fitness instructors. All service users were female, two of White ethnicity and one of Asian background. All participants spoke and understood English and could travel independently. One was a wheelchair user, two were mobile with a stick and orthotics/functional electrical stimulation.

During the event, discussions about barriers and facilitators to access physical activity and exercise options produced a number of themes. Service users told us: (i) it was unhelpful to be on an NHS waiting list for many months; (ii) they did not feel confident in a public gym because of the environment and lack of staff experience with regard to their needs, (iii) public gyms were too expensive, (iv) transport and access to gyms and equipment were a challenge. Service user views at the time of the event corroborated with service performance data from 2016 to 2017 indicating that people waited a mean (SD) of 49 (69) days (range: 2.5–57.5 days) to access NHS exercise groups often due to delays around waiting lists as well as with getting GP approval, a ‘fit note’, to exercise. Subsequently, only 8% of service users transitioned to local gyms, leaving 92% of service users unable to access ongoing physical activity and exercise options.

Service users suggested that to ensure confidence during transition from our NHS gym to public gyms in the future it would help to have: (i) a friendly face at reception, (ii) access to a dedicated fitness instructor at a quieter time in the day at a discounted rate, (iii) access to equipment for all abilities, (iv) physiotherapists providing extra support and education to gym staff; (v) a document to show the fitness instructor about their exercise ability and precautions.

This service user engagement was central to developing the NeuroActive pathway to ensure safe and effective transition from the NHS to local gyms and fostered self‐management around exercise and physical activity. Better Gym accommodated service users' requests relating to their staff and the gym environment (points i–iii). Physiotherapists provided extra support and education to Better Gym staff during transitions and developed a document supporting transitions (points iv and v).

#### 3.1.2 Co‐Evaluation

Nine out of 12 service users (75%) transitioned from NHS services in 2017: 67% to the new pathway and one service user to a falls group (8%). Four service users (two females, two males) who transitioned to Better Gym were interviewed by A.P. (44%). They reported feeling satisfied with the new pathway and more confident in managing their neurological condition. Shared decision making within this co‐production was at the centre of the development of the NeuroActive pathway as service users shared what needed to change so they could access and have higher concordance with exercise programmes.

Following this successful launch in 2017, access to the NeuroActive pathway grew in 2018 following the opening of a second Better Gym in the borough. After discussion with GPs, NHS physiotherapists and Better Gym, the requirement for the GP's ‘fit note’ to access local gyms was removed as this was causing delays to gym registration. Close working relationships with doctors in our borough facilitated conversations resulting in the agreement that physiotherapists were well‐placed to make decisions around safety to exercise in local gyms in the absence of acute or uncontrolled medical issues; moving forward, physiotherapists informed GPs about service users transitioning to Better Gym under their supervision. Instead of a ‘fit note’, a ‘health passport for physical activity’ was developed by physiotherapists and allowed service users access to discounted membership, as well as being a way to offer specific guidance for fitness instructors around exercise ability (points ii and v). To facilitate self‐management, service users and/or their carers signed the health passport for physical activity to confirm they understood: (i) the need to contact their GP in case of medical concerns; (ii) that they were aware of ‘red flags’ during exercise and (iii) that they knew they could access the NeuroActive NHS service for advice as required.

### 3.2 Aim Two: Evaluation of the Pathway (2019–2020)

#### 3.2.1 Service Users

Data were collected for 14 months until March 2020, when the service closed due to the COVID‐19 pandemic lockdown. All 35 service users who were referred started the programme. One service user left the programme after breaking his hip (unrelated to the class) and then re‐joined following recovery. Therefore, service user demographics summarised in Table 1 include 35 service users from 36 referrals received.

#### 3.2.2 Performance of the Pathway

**Figure 1:**
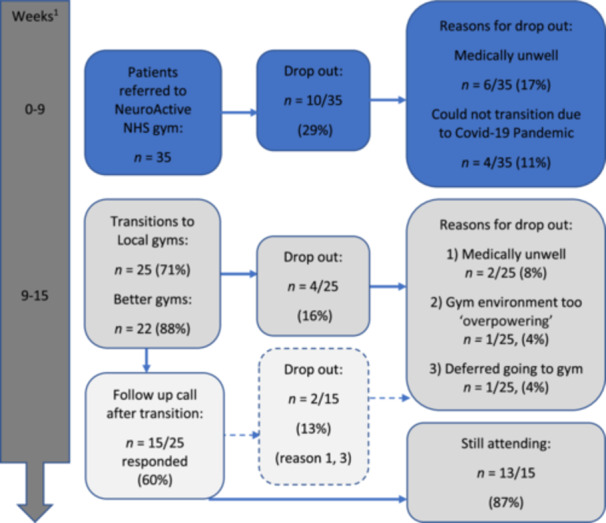
Number (*n*) of referrals and flow through the pathway. ^1^Most participants were in the NHS NeuroActive gym for < 9 weeks and transitioned to third sector gyms > 9 weeks.

In terms of service user flow, the mean (SD) response time from referral to NeuroActive to first assessment was 17 (15) days (*n* = 36). Figure 1 describes service user numbers at each stage of the pathway, including reasons for dropping out. Out of 35 service users, 25 (71%) transitioned to third‐sector gyms the week following discharge. The main reasons for not transitioning and dropping out were medical issues and the start of lockdown due to the pandemic, for four (11%). After transition two (8%) service users dropped out due to feeling unwell. Once transitioned, only one (4%) service user reported the gym was ‘overpowering’ and chose to join a smaller gym. Thirteen out of 15 (87%) participants who responded to the follow‐up survey were still attending a third sector gym 6 weeks after transition. No service users were referred back to our physiotherapy service.

Total length of attendance to the NHS NeuroActive sessions was mean (SD) 67 (31) days with 1.3 (0.5) 1‐h sessions per week. Service users attended mean (SD) 8.6 (4.8) sessions and missed an average of 0.8 (1.8) sessions.

#### 3.2.3 Feasibility of Service User Outcomes of the NeuroActive NHS Intervention

As seen in Table 2, there was a mean (SD) 0.8 (8.3) increase in score on the MOEES from entry to discharge. Systolic and diastolic BP reduced by mean (SD) 1 (16.2) and 5.1 (7.9) mm Hg, respectively. There was an improvement on the 10MWT normal and fast with an increase in walking speed of mean (SD) 0.8 (2.6) and 0.5 (1.5) s, respectively. The number of stands in 30 s (STS) increased by mean (SD) 0.7 (3.7) stands and hand grip strength for the right and left increased by mean (SD) 2.7 (3.8) and 4.7 (5) kg, respectively. There were trends suggesting improvements in walking speed, the number of sit‐to‐stands, and grip strength, but these changes were not clinically significant. Improvements in physical activity to a mean (SD) of 177.9 (420.6) min per week could be viewed as clinically meaningful as they meet physical activity recommendations of engaging in at least 150 min of moderate or 75 min of vigorous intensity aerobic physical activity each week.

Results in Table 2 are given alongside the number of service users completing that specific measure. Reasons for missing data per outcome measure were varied and are clarified in Table 3.

**Table 3:** Causes for missing data per outcome measure.

Missing data–Nr/35 (%)^a^	MOEES^b^	BP^c^	10 MWT n^d^	10 MWT f^d^	STS^e^	HGD r^f^	HGD l^f^	PAVS^g^
Total	**15/35 (43)**	**16/35 (46)**	**18/35 (51)**	**19/35 (54)**	**12/35 (34)**	**18/35 (51)**	**15/35 (43)**	**7/21 (33)**
Medical reasons	6/35 (17)	6/35 (17)	6/35 (17)	6/35 (17)	6/35 (17)	6/35 (17)	6/35 (17)	1/21 (5)
COVID‐19 pandemic	4/35 (11)	4/35 (11)	4/35 (11)	4/35 (11)	4/35 (11)	4/35 (11)	4/35 (11)	4/21 (19)
Early transition to local gym	1/35 (3)	1/35 (3)	1/35 (3)	1/35 (3)	1/35 (3)	1/35 (3)	1/35 (3)	1/21 (5)
Not appropriate for service user	3/35 (9)	0/35 (0)	5/35 (14)	6/35 (17)	0/35 (0)	3/35 (9)	0/35 (0)	1/21 (5)
Staff error^h^	1/35 (3)	5/35 (14)	2/35 (6)	2/35 (6)	1/35 (3)	4/35 (11)	4/35 (11)	0/21 (0)

^a^ Number of missing data (percentage) for each outcome measure.

^b^ Multidimensional Outcome Expectations for Exercise Scale (max score 75).

^c^ Blood pressure (mm Hg).

^d^ Ten‐metre walk test normal and fast (s).

^e^ Number of sit‐to‐stands in 30 s.

^f^ Hand Grip Dynamometry, right and left.

^g^ Physical Activity Vital Sign.

^h^ Occurred at the start of the evaluation for service users 1–16.

#### 3.2.4 Service User Outcomes After Transition to Local Gyms

Fifteen out of 25 (60%) service users responded to the follow‐up call, and 13/15 (87%) were still attending local gyms. Eight out of 13 (62%) service users completed the PAVS (at follow‐up) and had been physically active with a mean (SD) of 626 (642) min (IQR: 30–1365) over the last 7 days.

## 4 Discussion

The NeuroActive pathway facilitated access and opportunity to engage with physical activity and exercise programmes for service users with neurological conditions. Participants had various neurological conditions and levels of ability, often living with multiple co‐morbidities and very low activity levels (PAVS). Despite this, expectations for exercise (MOEES) were high, indicating service users believed they would benefit from accessing physical activity and exercise. Adherence was high, with 71% (25/35) completing the NHS phase of the pathway, and all service users transitioning to a local gym supported by a fitness instructor within 1 week of finishing NeuroActive. Furthermore, at 6‐week follow‐up, 87% (13/15) of service users surveyed were still attending a local gym. The main reasons for attrition were service users being medically unwell and the start of the COVID‐19 pandemic lockdown, rather than negative feedback about the pathway. Service user outcomes suggested beneficial trends in function, strength and particularly physical activity.

### 4.1 Context

This evaluation supports existing findings in the literature related to the beneficial effects of increasing physical activity and exercise [10, 13, 14, 15, 16, 17, 18, 19, 20, 21, 22, 23, 24]. The success of the NeuroActive pathway may be due to the co‐production element [32, 33, 34, 35, 36]. By including service users in the development stage, as was done for the FAME [30] and I‐REBOUND [31] programmes, it enabled the development of a pathway that addressed barriers to physical activity and exercise experienced by service users, similar to what is described in the literature [25, 26, 27]. The development of the NeuroActive pathway addressed some of these barriers by supporting service users in managing their neurological impairments until they could access a local public gym. Preparing service users in an NHS gym increased their function, strength and physical activity levels before transitioning to a public gym environment. The NeuroActive intervention is a formal programme delivered in small groups in which participants formed social networks that continued after NHS discharge in their local gym, another potentially important facilitator to physical activity and exercise participation, also recognised in the literature [28, 29]. A friendly face at the gym reception and a dedicated, trained fitness instructor were further important local changes. In France, although not started in the healthcare setting, a similar study [46] described how an adapted physical activity‐based programme was provided in a fitness centre for community‐dwelling adults with neurological conditions. Different outcome measures were used compared to our pathway, but 79 participants improved significantly on body strength measures (by 37%–49% measured on gym machines) and an aerobic measure (by 22% on the 6‐min walk test). Adherence was also good as 86% of participants completed the 6‐month programme and 83% subsequently purchased a 1‐year gym subscription. Our evaluation indicated adherence of 71% (25/35) completing the NHS phase, with 87% (13/15) of those interviewed still attending a local gym 3 months later. All service users in our evaluation became gym members at a low cost, although the Pandemic prohibited further follow‐up.

A qualitative study reporting on the lived experience of eight participants following stroke who exercised in a community venue found that it was possible to offer accessible exercise options for people with limited mobility [47]. The centre had a gym area comprising power assisted exercise (PAE) equipment: a machine offering multi‐directional assisted movements which enable the user to simultaneously engage all four limbs and the trunk whilst seated, a motorised treadmill, MOTOmed and static parallel bars. Participants associated the ability to use this equipment alongside venue membership as a turning point in their adjustment to life following stroke and long‐term recovery in third‐sector services. Apart from PAE, the same equipment was offered in the NeuroActive pathway. Similar referral options existed as well (either self‐referral or by a healthcare professional) and access to physiotherapy support. Participants were required to pay for their gym membership package themselves, and this may have been a barrier to attendance for people from economically disadvantaged households. This is why, in the NeuroActive pathway, access was offered at very low cost, inclusive to most, but potentially not for those who could only access free options.

Perspectives and experiences from Swedish and New Zealand physiotherapists in 2010 suggest that therapists have innovative expertise to support people with neurological conditions to exercise in recreational environments [48]. However, nine physiotherapists interviewed described constraints within healthcare and recreational areas that hindered the promotion of physical activity for this cohort. Despite policies supporting exercise, therapists perceived a lack of support from health care and a lack of knowledge of disability within the recreational area. This was also a theme identified during the development of NeuroActive. Ring‐fencing funding for the pathway and providing training to fitness instructors in local gyms from neurophysiotherapists may have contributed to the success in transitions and adherence to exercise following discharge from NHS services.

### 4.2 Limitations

A limitation of the evaluation was missing data, primarily due to service users being unwell rather than service users dropping out because they did not value the pathway. Most participants were living with multiple co‐morbidities as well as a (new) neurological condition, and this may have influenced the risk of being episodically unwell (1). The start of the COVID‐19 pandemic lockdown was the other main reason for missing data, however, only the last month of data collection was affected suggesting a minimal overall impact (4/35, 11%). The lockdown, however, eventually did stop all access to NHS and local gym services for 80% (28/35) of participants.

A second limitation concerned the reliability of the outcome measure protocol with data collected by clinical staff in a busy therapy gym setting, and this could have resulted in some operator error. Regular staff training and support around measurement and data entry likely maintained reliability and minimised staff error. It is, however, recognised that missing data due to staff error ranged from 3% for the MOEES to 14% for BP for the first 16 service users (some staff forgot to take BP). Following the first quarterly meeting with physiotherapists, this was addressed by staff training on the entire protocol. Service users, 17–35, had complete data apart from one missing data point on one measure. At this meeting, it was recognised that the PAVS would be a valuable outcome to collect for measuring physical activity levels. This means that no data on the PAVS was recorded for the first 16 service users. This measure was viewed as particularly valuable as it could be collected after transition, when no further data was collected by Better Gym staff.

The third limitation was the suitability of the outcome measure protocol. Although it was suitable for service users with a range of neurological conditions commonly seen in NHS neurorehabilitation services, as anticipated, not all outcome measures were appropriate for all service users due to specific neurological impairments. The 6‐min walk or push test was not used due to a lack of space in the gym, and the MOEES was not completed by some due to communication impairments. An inability or reduced ability to walk (wheelchair users) meant that some service users could not complete the 10MWT. Arm paralysis prevented service users from completing the grip strength measure. The measure that was completed the most together with the PAVS was the STS, as it included three service users who were unable to walk (10MWT) but could stand up. The STS measure is viewed in the literature as a responsive measure to assess function in people with neuromuscular disorders [41].

A cautious overall interpretation is that service users managed to increase their physical activity levels and adherence to their exercise programmes by taking part in the NeuroActive pathway. The pathway succeeded in improving physical activity outcomes and removing barriers around access, costs, safety, environment, equipment and support in local gyms, enhancing adherence to exercise. The transition sessions, supported by physiotherapists, fitness instructors and the provision of the health passport for physical activity, facilitated an efficient and safe transition to local gyms. It facilitated a clear message and assisted service users in attending, at a lower cost, in a safe and supported environment, addressing barriers raised during the engagement event.

In terms of generalisability, this evaluation was specific to a Central London service and borough. However, the general principles of facilitating physical activity and exercise are well‐evidenced and likely replicable in other community settings [6, 7, 8, 9, 10, 11, 13, 14, 15, 16, 17, 18, 19, 20, 21, 22, 23, 24] in the United Kingdom and internationally with similar contexts. Local third sector party collaborators, specifically Better Gym, Local Exercise Groups (LEGS) and the Stroke Association, also offer services nationally, allowing replication at a larger scale in the United Kingdom. Support for the pathway demonstrated that co‐producing sustainable services with local partners was possible. Increased access and adherence to physical activity and exercise benefitted service users and could potentially reduce health complications in the future, requiring (avoidable) referral to NHS services [1, 4, 49].

## 5 Conclusions

Outcomes suggested the pathway enabled service users to timely access and adhere to physical activity and exercise immediately following discharge from neurorehabilitation. This pragmatic service evaluation included small numbers and suggested trends in beneficial clinical outcomes in function, strength, and particularly physical activity levels. This evaluation could potentially inform: (i) future investigation at scale; (ii) development of the health passport for physical activity as a tool to facilitate faster access to more exercise settings for this vulnerable cohort and (iii) further co‐production to grow the pathway and secure strategic decision‐making in policies [32, 33, 34, 35, 36]. Future work on physical activity and exercise should investigate: (i) long‐term adherence to local exercise schemes and reasons why service users drop out, and (ii) an economic evaluation including, for example, the incidence of health complications, GP appointments and emergency department attendance. This could help to assess if some of the acute pressures on NHS services could be alleviated by accessing pathways such as NeuroActive that focus on increasing access and adherence to physical activity and exercise and reducing sedentary behaviour for service users with neurological conditions.

## Conflicts of Interest

Caroline Appel declares that NOCLOR, a research institute in London, England, provided a grant (£10.000) to backfill C.A.'s clinical role so C.A. could focus on data analysis and writing for publication. The other authors declare no conflicts of interest.

## Data Availability

The data that support the findings of this study are available from the corresponding author upon reasonable request.
